# What the Iberian Conquest Bequeathed to Us: The Fruit Trees Introduced in Argentine Subtropic—Their History and Importance in Present Traditional Medicine

**DOI:** 10.1155/2013/868394

**Published:** 2013-11-24

**Authors:** Pablo C. Stampella, Daniela Alejandra Lambaré, Norma I. Hilgert, María Lelia Pochettino

**Affiliations:** ^1^Laboratorio de Etnobotánica y Botánica Aplicada, Facultad de Ciencias Naturales y Museo, Universidad Nacional de La Plata, 1900 La Plata, Buenos Aires, Argentina; ^2^CONICET, Argentina; ^3^Laboratorio de Botánica Sistemática y Etnobotánica, Facultad de Ciencias Agrarias, Universidad Nacional de Jujuy, San Salvador de Jujuy, Argentina; ^4^Instituto de Biología Subtropical, CONICET, Facultad de Ciencias Forestales, Universidad Nacional de Misiones, CeIBA, 3370 Puerto Iguazú, Misiones, Argentina

## Abstract

This contribution presents information about the history of introduction, establishment, and local appropriation of Eurasian fruit trees—species and varieties of the genera *Prunus* and *Citrus*—from 15th century in two rural areas of Northern Argentina. By means of an ethnobotanical and ethnohistorical approach, our study was aimed at analysing how this process influenced local medicine and the design of cultural landscape that they are still part of. As a first step, local diversity, knowledge, and management practices of these fruit tree species were surveyed. In a second moment, medicinal properties attributed to them were documented. A historical literature was consulted referring to different aspects on introduction of peaches and citric species into America and their uses in the past. The appropriation of these fruit-trees gave place to new applications and a particular status for introduced species that are seen as identitary and contribute to the definition of the communities and daily life landscapes. Besides, these plants, introduced in a relatively short period and with written record, allow the researcher to understand and to design landscape domestication, as a multidimensional result of physical, social, and symbolic environment.

## 1. Introduction

The native human groups—diverse in patterns of subsistence and settlement strategies—that, since prehispanic times, inhabit different ecological territories of our country, constructed particular cosmovisions. Those cosmovisions together with local knowledge shaped the practices of management and use of those plant resources that defined the local landscape while designing a dynamic physical space where the own meanings and values of each people were manifested themselves [1, 2]. This process includes the features that define domestication (cultural selection and the emergence of a different reality), and its result is the construction of a cultural landscape that includes the dimension of biocultural phenomenon, in the sense that it is a physically, culturally, and historically determined environment [1, 3].

As of the process of European settling and peopling, like the Jesuit reductions and the founding of colonies in Argentine territory were, those cultural landscapes changed, as well as the characteristic ways of life of those ethnic group. So, the breakup of natives spaces occurred along with the establishment of a new regime of exploitation and production, either politically or culturally imposed by the Europeans, by means of the introduction of stuffs and products from the Old World, for instance, cattle and several crops that modified the configuration and representation of landscape [1, 4–7].

The settling of Spanish colonies in America led to the beginning of plant species exchange between the New and the Old World that would affect multiple aspects of daily life in both continents. Studies seldom approach the changes produced since 1492 in the particular features of each group in agriculture, food, and even in their habits and customs—as medicine, rituals, and religious celebration. Instead, there are many examples that refer to the expansion of plants since times much older than the arrival of Spaniards to America, and those cases show similar situations in other parts of the world, in particular the movements of goods and species from the East and the South (Africa) to the classical Mediterranean. Among them, “sugar cane” (*Saccharum* ×* officinarum* L.) can be mentioned which was rapidly adopted in the South of Europe through the advance of Islamic Empire [8]. As well, the European contact with Africa in the middle 15th century was partially promoted for the desire of acquiring spices and condiments. The silk route, established by the 1st and 2nd Century, constituted a very important commercial way for the exchange of cultivated spices. Plant species from the Old World arrived into America as part of culture and idiosyncrasy of Iberian people, for example, “wheat” (several species of *Triticum*), “barley” (*Hordeum vulgare* L.) and “rye” (*Secale cereale* (L.) M. Bieb.), “broad bean” (*Vicia faba* L.) and other pulses, seed and/or stone fruit trees of the gener *Prunus*, *Cydonia*, *Malus*, and *Pyrus* (“plum,” “peach,” “quince,” “apple,” and “pear”), and other temperate fruits, along with aromatic species and flowers that were included as exotic elements within American native communities [5, 9–11].

**Figure 1:**
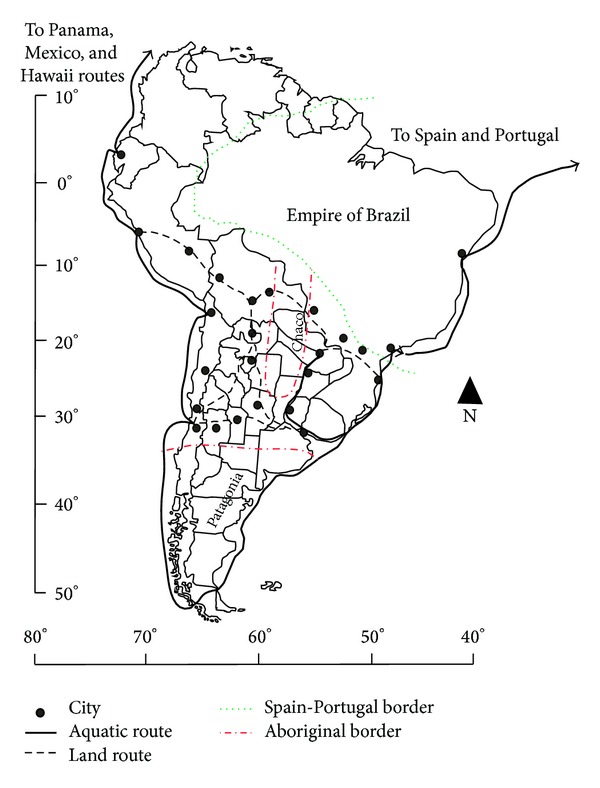
The Iberian conquest and colonization of America continent: an routes to terra firma of the 16th Century.

All kinds of vessels were constantly exchanging plant germplasm between both worlds, and besides seeds, fruits, and adult plants, the transportation of branches, shoots, and grafts was also frequent (“*ramas*,* retoños*, *varetas y mugrones (ingertos) de algunos árboles* …,” Anglería (1516) in de la Puente y Olea [12]), in barrels for wine. The main destination of these trips was Antilles and later the continent (which was named “tierra firme,” that is, terra firma), Mexico and Peru the (16th Century). Then, according to chronicles, different access routes were followed. This route, known as Caribbean-Andean, was accompanied by the one of Asunción-La Plata River and the Brazilian one. Each of them had its own particularities related to interethnic relationships, the origin of the introduced germplasm, and the associated knowledge with each plant and its management (Figure 1).

### 1.1. Fruit Trees and Local Medicine

Many cultivated plant species with medicinal properties arrived to America, along with associated knowledge that was propagated by Spanish people. Their incorporation and appropriation by local people involved a process of assignment of properties, the progressive perception, and the search among the alternatives of obtaining, producing and applying of their medicinal use.

De la Puente y Olea [12] gave the first references of these medicinal species that were also mentioned in the description of the orchards of the priests of two Jesuit missions given by Sepp [9] and Paucke [13]. Furthermore, several volumes about medicine and medicinal plants were found among Jesuits goods in the moment of their expulsion from America (Galeno de Pérgamo (the 2nd Century), Rivero, Johann Dolaeus (the 17th Century), and Suárez de Rivera, y Fuente Piérola (the 18th Century)), and it is supposed that they used also the books of Andrea Mattioli, Andrés de Laguna, Dioscórides and the Materia Medica of Father Joseph of Montenegro, and Father Segismund Asperger [14]. On the other hand, it is worth mentioning that all around the world, different human groups attribute medicinal value to their food, and in this way, the species considered in this contribution, which all have been originated in Asia or Eastern Europe, were added to the Mediterranean record of useful plants by their biodynamic properties as well: citric as insect repellent or antidote in poisoning and for diverse diseases and symptoms, while the fruit trees of rose family were valued as astringents, febrifuges, diuretics, and laxatives [15, 16]. In the particular case of peaches, they were appreciated as a luxurious food, and in so much, they appear also in funebrial contexts in the Mediterranean [17]. These therapeutical uses, even if they do not remain unaltered through the time, reflect a minor incidence of commercial aspects than their use as food and also show the local appropriation of these plants through the generation of novel applications.

Based on what is mentioned earlier, this contribution provides information about the medicinal use of two plant groups of Eurasian fruit trees in America, considering that their history of introduction, appropriation, and local recognition allows to interrelate them and to define them as representative elements of the landscape that they are currently part of. The two plant groups are part of larger scale studies and reflect the research performed by the authors on different periods and regions of Argentine subtropics: (a)—the case of citric, *Citrus* L., from northeastern Argentina and (b)—“peaches,” *Prunus persica* (L.) Batsch., from northwestern Argentina. From the ethnobotanical approach, the description, analysis, and comparison of the entry routes are presented, as well as the medicinal use of citric and fruit trees of rose family in the past and in the present. This kind of study allows to make a time projection with the historical data and ethnobotanical information to reach their appropriation significance.

### 1.2. Description of Local Landscape

#### 1.2.1. Northeastern Argentina (NEA): Phytogeographic Districts of “Campos” and “Urunday” in the South of Misiones

Field works have been performed in two departments of the south of the province, San Ignacio (in the Paraná river bank) and Concepción de la Sierra (near the Uruguay river). This zone is a transition between the District of Campos and the District of Selvas Mixtas [18], where Martínez-Crovetto [19] places the district of Urunday. In this region, gallery forests form corridors along Parana and Uruguay River and also along the course of the main streams, and they also cover the slopes of low hills as Santa Ana, San Juan, or San José. This is an impoverished forest, in transition to savanna, where forest islands within a grass matrix can be observed, which is locally known as “capones” [20]. The populations settled there are expressive of the history of the region. Diverse ethnic groups inhabited the area before the European arrived there, and since the beginnings of the 20th Century, several migratory waves have arrived. At present, a mosaic of different cultures can be found in the area shaping mixed communities: the “Criollos,” Mbya Guarani people and the descendents of Polish, Ukrainians, Italians, Spaniards, Brazilians, and Paraguayans, among others. The studied communities of Teyú Cuaré and Cerro Mártires are a good example of people diversity, and they can be defined as peasant communities [21]. This diversity is also reflected in the language as both Spanish and Guarani are spoken and also a local variation of Portuguese (in the Uruguay river area). As for the economy, local groups derived their subsistence from the “monte” (local name for the forest, [3]) through hunting, fishing, and gathering, and they also practiced a swidden horticulture of *Manihot esculenta *Crantz, *Ipomoea batatas *(L.) Lam., *Zea mays* L., and different species of *Phaseolus* and *Cucurbita*, along with other crops of low lands [22–25]. Native people living in the zone still carry out these activities, and they have added cultures introduced in the area by the Europeans, for instance, *Oryza sativa* L. and recently *Glycine max* (L.) Merr. They have also introduced livestock raising (cows, sheep, and minor animals). At present, the most important economic activities in Misiones are silviculture and agriculture, complemented with cattle raising. The first of them is based on monoculture of “pine” (*Pinus* spp.) and “eucalyptus” (*Eucalyptus* spp.) destined to paper and wood industries settled in the area, which are in turn job source for many inhabitants. The principal crops are “tobacco” (*Nicotiana tabacum* L.), “yerba mate” (*Ilex paraguariensis* A. St.-Hil.), “tea” (*Thea sinensis* L.), and fruit trees, mainly citric (*C. reticulata*, *C.* × *aurantium*, and *C.* × *latifolia*). In summary, the economy of the province is based in extractivism or production of raw material with low industrial development [26, 27]. In accordance with this scope, all the families interviewed in rural contexts are characterized by their traditional systems of subsistence production, with the occasional sale of surplus in urban tax-free fairs.

#### 1.2.2. Northwestern Argentina (NWA): Dry Valleys and Yungas (Cloud Forests)

**Figure 2:**
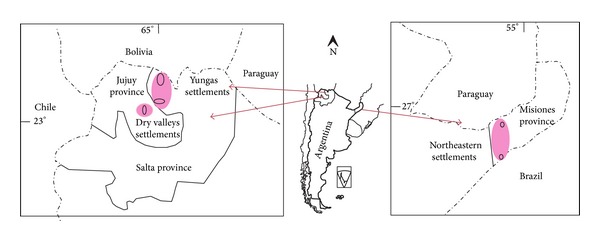
Localization of study areas in northwestern and northeastern Argentina.

Field works have been carried out in peasant communities of northwestern Argentina, settled in both western and eastern slopes of Andean foothills. In the west, the research was conducted in intermountain valleys in the localities of Juella and Yacoraite, in Humahuaca Ravine (province of Jujuy), and in the East in two areas in the province of Salta, in Los Toldos valley (Santa Victoria Department) as well as in the villages that are settled in the old Farm San Andrés (in Orán department) (Figure 2).

Western intermountain valleys correspond, from the phytogeographic point of view, to the Prepuneña or Bolivian-Tucuman province, characterized by a xerophytic vegetation, while the eastern slopes belong to Yungas or cloud forest, which are determined by the presence of orographic rainfall that define an exuberant vegetation, which varies according to the elevation shaping three different environments: Pedemontane Forest, Montana Forest, and Montane Cloud Forest [18, 28]. In spite of the marked differences resulting from the features of vegetation and general environment, in both regions agriculture is performed, destined not only to family consumption but also to the sale of products in fairs and regional markets. This subsistence strategy has a long history in the zone, where in addition to the typical American triada (*Z. mays*—*Phaseolus* spp.—*Cucurbita* spp.), several crops characteristic of Southern Andean Area were grown, for example, *Chenopodium quinoa* Willd.,* Solanum tuberosum* L., and other species of Andean tubers like *Oxalis tuberosa* Molina and *Ullucus tuberosus* Caldas, among others [29–31].

The inhabitants of these locations are descendents of native people; most of them with different degrees of miscegenation with Europeans arrived to the zone in different moments. The language there spoken is a local variant of Spanish, with the persistence of some words and grammatical structures of Kechwa and Aymara [32–35].

## 2. Materials and Methods

The present study is the result of a major ethnobotanical research conducted in different stages. In the first moment, the issue of agrobiodiversity richness and ways of management were approached, and then the aspects related to local uses of the fruit trees were studied in depth to obtain generalizations, in particular the therapeutic applications that are the basis of this contribution. In Yungas, field work was performed between the years 1994 and 2000, with the purpose to enquire about traditional medicine and phytotherapy in general. In dry valleys and northeastern Argentina, the researches started in 2010 and still continue. In both cases, the object of the study is the local knowledge and management of species and varieties of the genera *Prunus* and *Citrus*, respectively. Qualitative techniques have been used. The first settlers that took part in the research were randomly selected, and in some cases, the strategy of snow ball was applied [36, 37]. In NWA, the number of participants in the research reached in Yungas 59 people, in dry valleys 20 people, and in NEA 36 people. The interviews were oriented to the enquiry about local knowledge on the name or names by which the species are recognized, useful parts and allotted uses, with special reference to the therapeutical ones, including way of preparation and administration. Likewise, by means of the review of a total of 38 bibliographic sources and historic documents of different nature, ranging from the beginning of the 16th century to the present, the search for medicinal uses has been widened, and information about the entry of these crops in America was obtained. The uses of these ethnovarieties in the past and in the present were compared by means of chi-square test. Voucher and other support materials (like branches and leaves, as well as fruits) have been collected in the field in the company of local inhabitants. This plant material has been botanically identified [38–43], its taxonomy was controlled and updated [44, 45], and then it was deposited in the Herbary of Useful Plants and Collection of Fruits and Seeds (CFS) of the Laboratorio de Etnobotánica y Botánica Aplicada (LEBA) of the Facultad de Ciencias Naturales y Museo, Universidad Nacional de La Plata, and in the Herbary of Museo de La Plata (LP). They have been assigned the initial and number of personal records of each collector.

## 3. Results and Discussion

### 3.1. History of Entry Arrival and Use of Citrus in NEA

*Citrus* is native of east, south, and southeast of Asia, Australia, and southwest of Pacific Islands [44]. Most of citric species were introduced in Europe by Muslims during the 10th and 11th [46] with the only exception of “citron” (*Citrus medica* L.) that was already known by Greeks and Romans, and those fruits that were later introduced like “sweet orange” (*Citrus* × *aurantium* L.), “tangerine” (*Citrus reticulata* Blanco), and “grapefruit” (*C.* × *aurantium*). Citric were brought to America during the second trip of Columbus in 1493. The chroniclers of that period mentioned that seeds of “oranges,” “lemons,” and “citron”, as well as “melons” and all sort of vegetables (*pepitas y simientes de naranjas, limones y cidras, *(además de) *melones y de toda hortaliza *[47]), have been taken to Central America from the Gomera Island (that belongs to the Canarias archipelago). These references would correspond to *C. × aurantium *L., *C. × limon *(L.) Osbeck*, C. medica,* and *Cucumis melo* L., respectively. To these “species” (in strict sense, hybrid taxa or cultivated varieties [44]), de la Puente y Olea [12] added “toronjas” that would belong to *C. maxima *(Burm.) Merr., “limas” of the group of *C. × aurantifolia *(Christm.) Swingle, and *C. × limettioides *Tanaka. These authors also pointed out that the first routes of introduction of cultivated plants had been Andalucía and Castilla, in Spain; Guinea in Africa; and in Asia “orange” trees with large fruits which had been introduced from Filipinas. By the last quarter of the 16th century, several representatives of genus *Citrus*, as “oranges” and “lemons,” were already naturalized in the Americas. Father Joseph de Acosta (who arrived to Peru in 1572) mentioned that citric were the trees that had more and better developed, and he was surprised because of the presence of orange groves, as well as other citric including “limes,” “citron,” and similar (“*limas, cidra, and y fruta de este linage*” [48]). Those cultivars of *Citrus* that had been managed or domesticated in lesser extent, particularly those used as rootstocks for grafted plants, show a special easiness to naturalization, related to the environments where they have been planted, local people, fauna that eats their fruits, and their own process of hybridization and apogamy that are complementary of sexual reproduction [1]. It seems that the entry (as well as the exit) of plant germplasm was continuous and copious and more diversity appeared at the same time when the different areas of the world met in touch.

**Figure 3:**
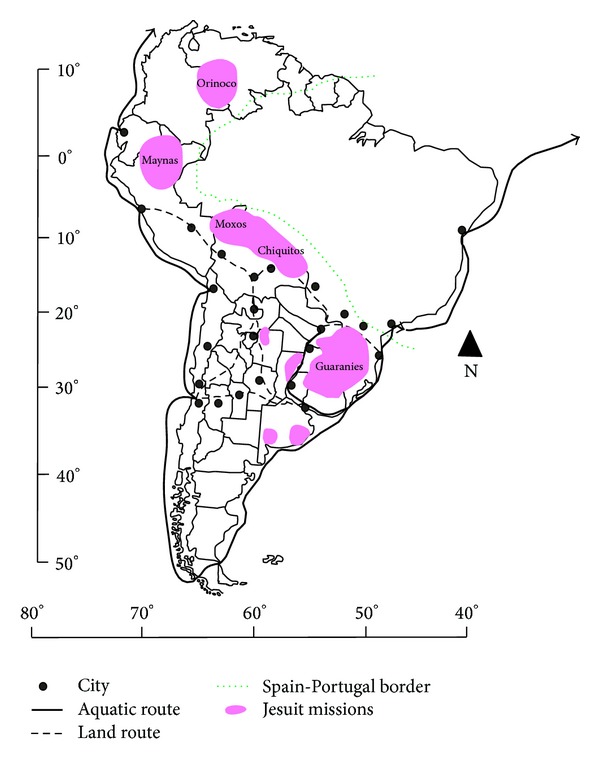
The result of the Iberian conquest and colonization of America continent: Latin American Jesuit Missions (the 17th and 16th Centuries).

During the Jesuitical period, citric were cultivated in different plots within the Guarani missions (Figure 3). The major diversity of fruits was found in the gardens of the priests and in the colleges [1]. In addition, there were “orange” groves belonging to the *Tupambae *(that were those community gardens for the production in large scale) destined not only for local consumption but also for the sale [49, 50]. The fruits were used in medicinal preparations against putrid fever (typhus), intestinal parasites, and stomach diseases, as appetizers and as antidotes against snake bites and other poisonous animals. The juice of the acidic varieties was considered to have cold properties in the frame of humoral medicine, and they were also uses as excipients and sweeteners, for instance, the syrup and bark of “citron” or “lemons” (*C. × limon*). Orange wine is cited as the most healthy of all liquors, and it is used as other spirits distilled from fruits, as a remedy for coldness in native people. This wine was made with orange juice fermented in big glass bottles with sugar, added with wine yeast fifteen days later and left to stand for a whole year [51].

“Orange” flower water was used (and it still is) in confectionery and the “bergamota” water (*C. × limon*) in perfumery [9, 52, 53]. After the Jesuits expulsion (1767), other uses are mentioned as, for instance, the use of bitter “orange” peel (*C. × aurantium*) to calm the ache of dysentery and as a tonic too; the juice of *“*lima sutí*”* (*C. × aurantifolia*) to stop paludic fever (“chucho”) and against the rush called “sudamina” o “zarpullido”. Nevertheless, some of these properties have been already discussed by Piso in the 16th century [54].

As from the contribution of Paucke [13] in the inventories of the Jesuits expulsion made by Brabo [55], a record of the cultivars grown in Jesuitical missions can be approached. There were cultivated “lemons,” “sweet lemons,” “limones ceutíes,” “sweet limes,” “citrons,” “toronjas,” and “sweet and sour oranges,” including cultivars like “Chinese orange” and others “oranges with fine peel.” Selected varieties were often cultivated in orchards and gardens of Jesuits, where only the priests and a few chosen Guarani people, particularly skilled for horticulture, were allowed to enter. They were also grown in the colleges of the order placed in Buenos Aires, Córdoba, Santa Fe, and Asunción del Paraguay. The cultivars considered that commons or vulgar were frequently cultivated in the *Tupambae* and occasionally in several *Abambae*—plots assigned to each family for subsistence production. Additionally, those varieties locally considered as “wild” grew spontaneously, in process of naturalization, in different spaces more or less modified by agroforestal management.

With the estrangement of the religious order from Spanish colonies in 1768, missions were abandoned and during the following year, multiple migrations occurred to the neighbouring forest, to Corrientes, Santa Fe, Buenos Aires, and Asunción, among other places [50]. Gardens were abandoned and a process of forest regeneration took place in several patches, formerly cultivated. It had to be expected that these anthropogenic landscapes were managed, especially those closer to buildings—some of them inhabited for a short period, despite the Portuguese and Paraguayan invasions in 1817 and 1818, in particular, fruits, *yerba mate*, and cotton (*Gossypium* sp.) groves, no longer functional. Orange groves still remain by the interviewed settlers as present a few years ago, and they have been described and praised by the travelers in Misiones during the end of the 19th Century and the beginnings of the 20th [56–60].

### 3.2. History of Arrival and Use of “Peaches” in NWA

*Prunus persica* (Prunoideae) is a species belonging to the botanical family Rosaceae. The genus is subdivided into several subgenera according to the morphology of the fruit. Based on bibliographic record, its origin can be established in the mountainous zones of Tibet and southwest of China. Peaches could be found in the Mediterranean by the principles of Christian area; nevertheless their taxonomy and history of evolution under domestication are still not completely elucidated as for origin and dispersal centers [61, 62].

The first galleons and other vessels coming from Sevilla arrived at the Antilles early in the 16th Century. They brought among other fruits (as mentioned previously) the fruits from the *Castillas* or stone fruits among which a special reference to “peaches” is made. Later, this germplasm entered Mexico and Peru (mainly in Lima), and from these points, the crop was expanded to the rest of the continent [12].

In the specific case of the populations of NWA, historical sources and records show that these exotic fruit trees were introduced from Chile—following the route of the Pacific—by the decade of 1550 by the expedition of Nuñez de Prado. They thrived and by December 1552 a wealthy fructification was described (“*ya surgían exuberantes los frutos de las primeras siembras de los españoles*” [63]).

The first seeds possibly arrived to Santiago del Estero, which was equidistant in the route of communication between Peru and the Atlantic Ocean and from there distributed to other provinces that were part of the colonial government of Tucuman, created in 1563 and constituted by the present Argentine provinces of Jujuy, Salta, Tucumán, Catamarca, Santiago del Estero, and the center of Córdoba. During this period, the influence of Chile on this government stopped and the district remained under the authority of Peru viceroy. The political space of Tucumán was born as a consequence of the discovery of Río de la Plata Litoral and the exploration of the route that connect it with Peru. Its occupation was guided by the need to expand the territory and to find new lands suitable for cultivation. Unlike the native groups that inhabited the south of Argentine territory, the Andean partialities that were comprised in this government have been soon subjected to the new administration after the arrival of the Spanish founders. Such was the case that by the second half of the 16th and beginning of the 17th Century, native people managed European crops, although these plants did not integrate their daily diet [4, 5, 63–65].

By that moment, in Argentina, the subsistence economy was based on the exploitation of native workforce to labor in the country with plants and animals introduced from Europe, with some local exceptions like “maize.” In 1590, a process of internalization of commercial circuit occurred, which became an integrated market from Potosi to Buenos Aires passing through Tucumán. Thus, the influence of Chile on NWA region was relegated [66]. In this frame, Jujuy was considered during colonial period a pathway employed by natives and Spaniards to arrive to Peru, and it constituted a border of Tawantinsuyu (Inca Empire) and later a pathway to Bolivia (Potosí) [67].

Along with the food use of peaches, there are archaeological evidence of their ceremonial use in ritual contexts by native people during Spanish-Indigenous period by the year 1600 [68]. Regarding their medicinal applications in the zone, the earliest mentions date from colonial period and referred to the use of peaches as natural laxatives. In fact, in different publications referring to European uses of “peaches,” the flowers and leaves are mentioned as to be used in infusion and/or syrup as child laxatives. Nevertheless, it is warned that flowers could be toxic because of the presence of amygdaline, more abundant in some varieties than in others [43, 69, 70]. Poultices with the leaves on the abdomen are recommended against parasites, as anti-inflammatory, and to heal wounds and herpes. The infusion of leaves with milk is also mentioned as antiparasitic. The distilled water of flowers is used to remove specks from the skin. Dry and powdered leaves are used to heal grains and furuncles and mashed seeds with egg white to stop hemorrhages [71].

### 3.3. The Current Situation of Citrus

By the end of the 19th Century, Burmeister [71] referred to the presence of “naranjos mandarinos” (tangerines, *C. reticulata*) in the province of Corrientes, and he recommended their intensive cultivation together with “common oranges” and “chirimoyo” (*Annona cherimola *Mill.) to export the fruits to large cities. A few years early, Hieronymus [72] included them in his publication about medicinal plants of Argentina under the name of “naranjo fino” o “mandarino” (before *Citrus deliciosa *Tenore, now *C. reticulata*) with the same uses than “sweet orange” (against scurvy, antitussive, and against bile diseases). “Pomelos” or “grapefruit” (*C*. × *aurantium*) entered possibly by the same time or shortly before than “tangerine.” It is still discussed if “grapefruit” is a result of mutation or hybridization, but there is agreement to consider Barbados as the place of geographic origin by the principles of the 18th Century, from where it disperses to the other Antilles islands not before a Century later [73, 74]. Maybe “grapefruits” were mistaken for “pampelmuse” or “toronjas” (*C. maxima*). These new introductions were coincident with the reconfiguration of local people through the migratory process that had place in the first years of the 20th Century. The first immigrants were brought to Misiones by official colonization that started in 1898 with the arrival of Polish and Ukrainians from Galitzia (one of the poorest zones of rural Europe) to the village of Apóstoles. The state was also responsible for the peopling of those lands of dorsal central ridge (Além, Oberá, and Cainguás). On the other hand, private colonization incorporated mainly Deutsch immigrants from Brazil or directly from Europe, and it covers mainly the zone of Upper Paraná. Later, Creole and Paraguayan immigrants arrived looking for job [75].

New wisdoms and practices on the environment arrived with the immigrants, as well as a different valoration of the plants with which they interacted; for example, the “oranges,” that in the new land were very common, in the origin countries were such a precious good that they were given as a present for Christmas. Another source of variability appeared with the industrialization of citric cultures. After the first quarter of the 20th Century, coincident with the start of citriculture in NEA, diverse cultivars were introduced from different parts of the world (Africa, India, USA, China, and Japan). The model of citric industry is the one developed in the states of Florida and California (USA) that consisted of new varieties grafted on rootstocks of “sour orange,” which was later replaced—because of CTV (*Citrus tristeza* virus, a kind of *Closterovirus*) epidemy—by rootstocks of “sweet orange,” “Cleopatra orange,” “*Rangpur lime*,” and “sweet lime”, among others [76–79]. By the decade of 1970, a homogenization took place because of the increasing agrarian globalization and market request for uniform fruits as for size, shape, colour, and taste. At present, 78% of cultivated citric belong to “tangerines” (“Satsuma,” “Clementina,” and “Murcott”), 17% to “oranges” (“Valencia Late,” “Salustiana,” “Lanelate,” and “Newhall”), and the remaining 5% to “lemons” (“Tahiti” and “Eureka”) [80]. In spite of this, several cultivars—some of them in a naturalized status [81, 82]—are conceived by most of the interviewed people as native fruit trees. “Apepú,” “naranja común,” “mandarina común,” “lima dulce,” and “limón mandarina” are considered wild fruits (from the “monte”). All the informants agree in considering “apepu” as native and wild, belief strengthened by the fact that this plant has a Guarani name. “Apepu” has different interpretations: some authors consider it formed by the prefix *a* meaning fruit, *pe* that is peel, and *pu* meaning noise and breakup [83]. Others [84] instead consider that “apepu” means flaccid, but *ape* would be dorsum or surface and *pu*, hollow sound, in reference to the noise made by the epicarp when knocked with the fingers, a distinctive feature of this fruit.

As a result of this research, 8 ethnospecies of *Citrus* (including 27 ethnovarieties) have been recorded which are summarized in Table 1. Local ethnovarieties are those generally named as “communes,” “caseras,” and “silvestres” (that mean common, domestic, and wild, resp.) and are marked in Table 1. They are coincident with those historically cultivated by the “Criollos” and can be found in the monte and also in old houses made of perishable material, known as “tapera” (that means abandoned house).

### 3.4. The Current Situation of “Peaches”

Early introductions of seed and stone fruit trees of the rose family resulted in the establishment of local cultures, which have been maintained by means of both seed or vegetative reproduction, but without the introduction of new germplasm. For that reason, those fruit trees populations are constituted by the same varieties introduced in times of the Colony, but they reflect local criteria of cultural selection as well as plant adaptations to a particular environment. In the case of “peaches,” those 500 years of history in the new settlement shaped a complex of 9 ethnovarieties comprised within 2 groups of ethnovarieties (Table 2) locally identified by diverse attributes that diverge from those prized by the market [85]. In this way, and although in the last years commercial “peaches” cultures were intended, in contrast to what happens with the citric in NEA, here the industrial cultivation of “peaches” has not been established, and the commercial varieties introduced did not represent an instance of diversification or entry of novel germplasm to local crop. The permanence is observed too in food and therapeutical uses given to these plants. Many inhabitants of dry valleys make a preserve (“compota”) with dry “peaches” (“pelones”) boiled with sugar, and the juice of the preparation is used to treat kidney diseases [86]. In Yungas, settlers cultivate “peaches” in the middle stage of the forest. There, a decoction made of dry flowers and powdered seeds is made to treat diseases related to intestines (diarrhea, flatulence, and intestine inflammation). The preparation is drunk three times a day, and if discomfort still persists, it can be taken for two consecutive days. The shoots are used in humoral medicine and soaked in vinegar, in frictions to remove heat from the body, or in infusion that has to be drunk three times to stop “chucho” (very high fever with sweat) [87]. The pulp of the fruit with apple pulp is mixed and mashed to use as a thickener element in the preparation of “yista”: a solid mixture of plant components with basic properties used to chew “coca” (*Erythroxylum coca* Lam.) [88, 89].

**Figure 4:**
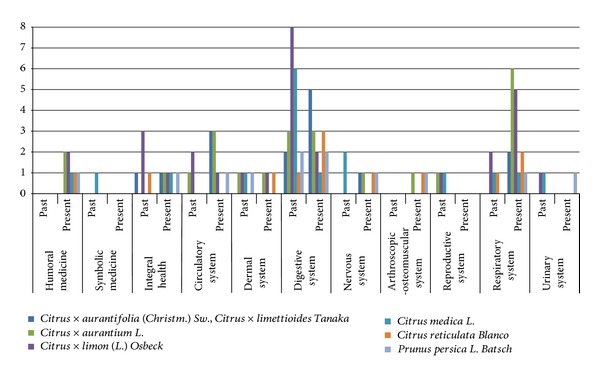
Number of therapeutic properties assigned per species per system.

**Figure 5:**
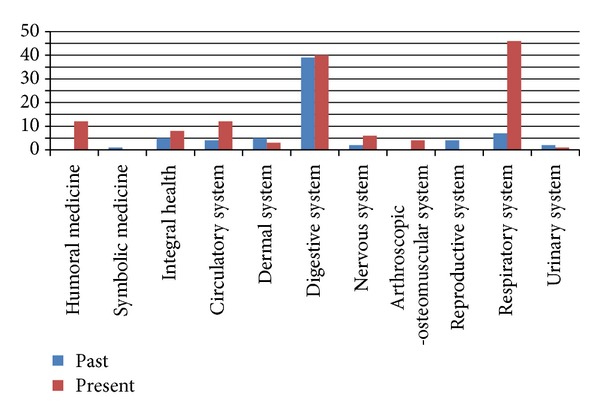
Number of ethnovarieties used for each body system in the past and present.

The information referred to the use of the previously mentioned ethnovarieties in the past, according to the literature, as well as the recorded uses in the study area at present, is detailed in Table 3 and compared in Figure 4. By comparing the number of ethnovarieties used for each body system either in the past and in the present, higly significative differences (*χ*210 g.l., *P* < 0.005 = 41.57) appeared (Figure 5). In Figure 5, it can be observed that the uses for affections of digestive system are the most frequent in both periods and that nowadays the usage of these resources becomes more important in the treatment of diseases of respiratory system as well as the circulatory, osteo-artro-muscle ones and humoral medicine.

## 4. Final Considerations

Characteristics of native people and of colonization streams shaped cultural landscapes with particular traits that reflect on the identified plant species and their uses. Among the fruit trees introduced in the northern Argentina, peaches had a long history of use in Europe (although their centre of origin was Asia). When peaches were brought to the American continent, during the early conquest and colonization, they were introduced as luxurious foods, reproducing their history in Europe, where they had a reduced number of therapeutic applications. The citric, instead, by that time had a short history shared with European people, and they were much valued not only as food but as medicines too. In NWA, the colonizers introduced cultivated plants from Spain and entered from Peru or Chile, though some contacts with the introductions performed through Buenos Aires port (over Río de la Plata) could be mentioned. From the ecological point of view, Humahuaca Ravine and other dry valleys were good recipients of “peaches,” “apples” (*Malus domestica* Borkh.), “pears” (*Pyrus communis* L.), and “quinces” (*Cydonia oblonga* Mill.). Consequently, both for cultural and environmental reasons, the local agricultural communities adopted rapidly these plants to cultivate them. But, along this process, new varieties consistent with local values had been selected, as well as those that showed more tolerance for the conditions in the new space. Thereby, varieties have been configured which are considered as defining a particular agricultural context and recognized as an identity factor by the settlers: “duraznos de la Quebrada” (in reference to Humahuaca Ravine). In NEA, on the other hand, the introduction of citric was the result of the confluence of two colonization streams: the Spanish one known as Río de la Plata-Asunción and the Brazilian one that summarized good apportations from Portugal, Africa, India, and the southeastern of Asia. Such citric, through the agency of Jesuits, became conspicuous elements in local landscape and turned into characteristic cultures of the zone. Furthermore, several taxa have passed to a naturalized status, and now they characterize also the spontaneous flora and are considered by the inhabitants as native from the zone.

In both areas, NWA and NEA, in parallel to the establishment of these crops as an agricultural and economic resource, diverse use has been developed, for example, the therapeutic applications referred to throughout the text, and many of them are novel with respect to those uses recorded in other parts of the world, as a result of local experimentation. Either past or present uses, although diverse, seem to be founded on the antioxidant properties of the fruits here considered, taking into account that oxidative damage, caused by the action of free radicals, may initiate and promote the progression of a number of diseases such as the ones treated with citric and peaches.

Despite the differences, the examples here posed allow to understand the processes of local appropriation of introduced plants. The decision to incorporate foreign elements has to be understood in the frame of the own cosmovisions of native groups from different zones of Argentina. Those groups were going through a moment of strong transformations, but they did not resign their prominence in decision making which contributed to cultural reproduction. Indeed, they took an active part, either incorporating as rejecting, redefining, or abandoning customs, objects, and products within the unceasing social and historic dynamics. In this cultural frame, the appropriation of “peaches” and citrus turns these originally exotic fruits into identifying elements that contribute to the definition of the communities and the landscape where their daily life has place. Given that those environments can undergo changes and so their material nature frequently varies through time, these plants introduced in a relatively short period and with written record allow the researcher to understand and to design landscape domestication, as a multidimensional result of either physical, social, and symbolic environment [108, 109].

## Figures and Tables

**Table 1:** The citric of NEA: local varieties and their botanical identity. The ∗ indicates the presumable local ethnovarieties.

Local name	Local variety	Botanical species	Voucher
Cidra	Cidra	*Citrus maxima *(Burm.) Merr.	Stampella 34 (LP)

Pomelo	Pomelo cidra	*C. maxima *×* Citrus *×* aurantium *L.	Not collected
	Pomelo blanco*	*C. *×* aurantium *(grupo pomelo)	Stampella 19 (LP)
	Pomelo rosado	*C. *×* aurantium *(grupo pomelo)	Stampella 78 (CFS)

Apepú	Apepú silvestre*	*C. *×* aurantium *(grupo naranja amarga)	Stampella and Hilgert 10 (LP)
	Apepú casera*	*C. *×* aurantium *(grupo naranja amarga)	Stampella 85 (CFS)
	Apepú dulce*	*C. *×* aurantium *(grupo naranja amarga)	Stampella 53 (CFS)

Naranja	Naranja silvestre*	*C. *×* aurantium *(grupo naranja dulce)	Stampella 128 (LP)
	Naranja silvestre de fruto grande	*C. *×* aurantium *(grupo naranja dulce)	Stampella 162 (CFS)
	Naranja casera*	*C. *×* aurantium *(grupo naranja dulce)	Stampella 112 (CFS)
	Naranja injertada	*C. *×* aurantium *+* Citrus trifoliata *L.	Commercial cultivar
	Naranja de ombligo (injertada)	*C. *×* aurantium *“Ombligo”+* C. trifoliata *	Commercial cultivar
	Naranja calderón	*C. *×* aurantium *“Calderón”	Commercial cultivar
	Naranja que guía	*C. *×* aurantium *	Not collected

Mandarina	Mandarina silvestre*	*Citrus reticulata *Blanco “Común de Concordia”	Stampella 103 (LP)
	Mandarina casera*	*C. reticulata *“Común de Concordia”	Stampella 121 (CFS)
	Mandarina injertada (con lima)	*Citrus reticulata* “Okitsu” (=*C. unshiu*) + *Citrus *× *limettioides *Tanaka	Commercial cultivar
	Mandarina injertada (con limón cidra)	*Citrus reticulata *“Encore”+ *Citrus *× *limon *(L.) Osbeck (=*C. jambhiri* Lush.)	Stampella 116 (LP)
	Mandarina injerto con apepú	*C. reticulata *“Encore”	Stampella, Cabanillas, and Hilgert 172 (CFS)
	Mandarina colorada o mandarinola	*C. reticulata “*Cleopatra” (=*C. reshni *Tanaka)	Stampella, Cabanillas, and Hilgert 170 (CFS)
	Mandarina colorada japonesa	*C. reticulata *“Okitsu”	Stampella, Cabanillas, and Hilgert 175 (CFS)
	Mandarina bergamota	*C. reticulata* “Encore”	Stampella 86 (CFS)

Limón	Limón o limón común*	*C. *× *taitensis *Risso (=*C. limonia* Osb.)	Stampella 80 (LP)
	Limón amarillo	*Citrus *× *limon *“Verna” (=*C. limon*)	Commercial cultivar
	Limón lima	*Citrus *× *latifolia *Tanaka	Stampella, Keller, Núñez, and Dutra 60 (CFS)
	Limón cidra o limón rugoso	*Citrus *× *limon *(L.) Osbeck (=*C. jambhiri*)	Stampella, Hilgert, and Furlan 132 (CFS)
	Limón sutíl o lima ácida	*Citrus *× *aurantifolia *(Christm.) Swingle	Not collected
	Limón real o limón aromático	*Citrus *× *limon *(L.) Osbeck (*C. *× *latifolia *× *C. maxima*?)	Stampella, Cabanillas, and Hilgert 177 (CFS)

Lima	Lima, lima dulce	*Citrus *× *limettioides *Tanaka	Stampella 79 (CFS)

Quinoto	Quinoto	*Citrus japonica *Thunb. (=*Fortunella japonica *[Thunb.] Swingle)	Stampella, Hilgert, and Furlan 150 (CFS)

*Trifoliata *	*Trifoliata *	*Citrus trifoliata *L. (=*Poncirus trifoliata *Raf.)	Not collected

**Table 2:** Ethnovarieties (locally recognized varieties) of peaches (*Prunus persica*) from dry valleys in NOA.

Group of ethnovarieties of durazno (*Prunus persica*)	Ethnovariety	Voucher
Durazno común (endocarp adhered to mesocarp)	Amarillo entero	22 Lambaré (CFS)
	Amarillo corazón rojo	31 Lambaré (CFS)
	Blanco	52 Lambaré (CFS)
	Rosado	45 Lambaré (CFS)
	Durazno jorge (=Cholo Cholito)	30 Lambaré (CFS)
	Durazno alancate (=Olancate)	60 Lambaré (CFS)

Durazno prisco (=Frisco that can be opened) (endocarp not adhered to mesocarp)	Amarillo	46 Lambaré (CFS)
	Blanco	50 Lambaré (CFS)
	Rosado	20 Lambaré (CFS)

**Table 3:** Summary and comparison of uses of the studied fruit trees. Past uses in Europe and present ones in study area.

Common name	Scientific name	Uses in the past		Present uses in the study area		Validation
		Part of the plant used	Medicinal Use [15, 16, 43, 47, 52, 69–71, 73]	Part of the plant used	Medicinal use	
Cidra	*Citrus medica* L.	Fruit	To avoid evil spirits, antidote against poisons (emetic), insect repellent, to freshen up breath, remedy against plague, to invigorate stomach and against vomiting and fainting after labour			Antiulcer [90]
		Juice	Effective purgative to remove poison from the body			Fever [47]
		Epicarp	Sedative			
			For halitosis, for dysentery, to comfort stomach, diuretic, and mild antidote	Epicarp	For the stomach, stomach ache, for diarrhea, against “empacho,” as tonic, and for the fever	Tonic, antioxidant, hypoglycemic, and anticholinesterase [47, 91]
		Leaf	For the stomach, tonic, stimulant, and expectorant			
			Antispasmodic	Leaf	Refreshing (against heath, in humoral medicine)	Fever, anthelmintic [47, 92]
		Shoots	After the labour			
			Appetizer, for stomach ache, and anthelmintic			
		Flowers	Medicinal			
		Seeds	Anthelmintic			Anticancer activity [47]
		Fruit	Antidote against poisons			
			Appetizer, to invigorate stomach, and antidote against poison			Antimicrobial, antimycobacterial and spasmolytic [47, 93, 94]

Lima	*Citrus *× *aurantifolia* (Christm.) Sw. *Citrus *× *limettioides *Tanaka			Leaf	Digestive, for stomach ache, for flatulence, and for gastrointestinal diseases	Antibacterial [47]
		Juice	To clean liver and stomach, to alleviate stomach inflammations, to alleviate fevers, to improve blood, and to cure wounds, throat, and uvula abscess.	Juice	To chew coca (dietary supplement)	Immunomodulatory effect [47]
				Fruit	To low blood pressure	
				Leaf	To low blood pressure, sedative, for the heart, and for diabetes	
				Flowers	For the heart	
				Juice	To low blood pressure, for diabetes, for the liver, sedative, digestive, for influenza, and for colds	
				Juice	Refreshing, for liver, stomach ache, against indigestion, for fever, for blood, for cough, expectorant, for influenza, for pneumonia, bronchitis, colds, angina, purgative, as anti-inflammatory in insect bites, aphthas, for hangover, and for lose weight	Antidiarrhoeic, diuretic, intestinal mucosa protector, local haemostatic, vascular stimulant and protector, vitaminic, antioxidant, sedative, and anxiolytic [47, 95]

Limón	*Citrus *× *limon *(L.) Osbeck	Seed	Against sickness and vomits			
			To prevent drunkenness			
			To eliminate pimples and blackheads			
			Anthelmintic and to eliminate kidney stones and sand			
			To relieve thirst and febrifuge			
			Antidote against poisons			
		Epicarp	To invigorate stomach, appetitive, digestive, to improve breath, to strengthen heart, antidote, to improve hygiene, and to remove phlegm from the palate			Antiseptic, carminative, diuretic, eupeptic, vascular stimulant, protective vitamin, and antimicrobial [47, 96]
		Fruit	To cure the plague and digestive	Fruit		Analgesic, antianemic, antiemetic, antiesclerotic, antipyretic, antiseptic, demulcent, moisturizing, remineraliser, antitoxic, and vulnerary [47]
				Flower	For influenza, sudorific, for colds, and for sore throat	
				Leaf	For influenza, for cough, and to low blood pressure	
		Juice	To smooth the face and to remove yellow color of jaundice	Juice	Dietary supplement, refreshing, sedative, digestive, and for respiratory diseases	Peel antiperoxidative, antithyroidal, anti- hyperglycemic, cardioprotective, anthelmintic, and antimicrobial [97, 98]

Naranja	*Citrus *×* aurantium *L.	Flowers	To invigorate stomach, heart, and stomach tonic and fainting after labour			
				Leaf	For flatulence, refreshing (humoral medicine), for hemorrhages, to low blood pressure, for influenza and fever, for cough, sedative, digestive, hepatic, laxative, angina, antiseptic, bone pain, and low back pain	
				Fruit	Sudorific and for influenza	
				Epicarp and mesocarp	For influenza, cough, and for colds	
		Flowers	To strengthen heart and stomach			Antispasmodic, sedative and tranquillizer [47]

Naranja agria, apepu	*Citrus* × *aurantium* L.	Epicarp	To comfort stomach	Epicarp and mesocarp	Fever, for influenza, digestive, and for hangover	Appetizer, cholagogue, demulcent, eupeptic, reduces cholesterol, tonic, vascular stimulant, it aids in digestion and relieves flatulence, cardiovascular health, anticancer, sedative, anxiolytic, and antiviral [47, 99, 100]
				Fruit	For influenza and for fever	
				Leaf	To low blood pressure, for influenza, digestive and for “empacho,” for “pasmo” and column “pasmo,” bone pain, hemorrhoids, and angina	
				Juice	Refreshing, vitamin supplement, for influenza, for respiratory diseases, cough and bronchospasm, for “pasmo,” and angina	
		Juice	For liver, cough, and against scurvy	Juice	Refreshing, digestive, and sedative	Antimicrobial [101]

Mandarina	*Citrus reticulata* Blanco			Sprout	For influenza	
				Leaf	Sedative specially for children, fevers, influenza, for cough, emetic, antiseptic, tooth ache, and waist ache	
		Leaf	For liver and antidote	Leaf	Against influenza, fever, for cough, and to low high blood pressure	Hypotensive, antistress, against anxiety, hypoglycemic, and hypolipidemic [102–104]

Pomelo	*Citrus *× *aurantium *L.			Juice	Vitamin supplement, refreshing, for cough, catarrh and asthma, influenza, digestive, and to low blood pressure	
				Epicarp and mesocarp	For fever	
				Fruit	Vitamin supplement and for influenza	
		Fruit	Laxante	Fruit	Kidneys and sight	Anti-inflammatory [105]

Durazno	*Prunus persica * L. Batsch	Leaf	Laxative, antiparasitic, anti-inflammatory, and healing			Laxative [106]
		Flowers	Laxative and to remove freckles	Flowers and seeds	Intestine diseases (diarrhea, flatulence, and intestine inflammation)	Skin diseases [107]
		Seeds	Against hemorrhage			
				Shoots	Refreshing and febrifuge	
